# Vaccination and Re-Vaccination

**Published:** 1864-01

**Authors:** 


*Vaccination and Re-Vaccination.*—The increasing prevalence of Smallpox and Varioloid throughout the country is a sufficient reason for calling the attention of the profession, and through it, that of the public, to the importance of attending to *Vaccination* and *Re-Vaccination.* There is reason to believe that many persons have suffered unnecessarily, by reposing in a false security, they having been subjected to spurious vaccinia. The following "conclusions" enumerate the conditions, and the protective influence of a perfect vaccination:

In 1845, the Academy of Sciences of Paris published a Report, containing the following conclusions: 1. The protective influence of vaccination is complete as regards the great majority of the vaccinated, and temporary as respects a small number only; and in these latter it is almost absolute up to the period of puberty. 2. Smallpox rarely attacks the vaccinated before the age of ten or twelve; and it is from this age to thirty or thirty-five that they are chiefly exposed. 3. That in addition to its preservative influence, vaccinnation endows the constitution with an influence which renders the symptoms of small-pox much milder and of shorter duration. 4. Cow-pox, directly or recently derived from the cow, is attended by a much more intense local phenomenon, and by a more certain and permanent effect than that which has passed through a great number of human subjects, this intensity of local action subsiding after many successive vaccinations. 5. The preservative influence of vaccination appears not to be intimately connected with the intensity of its local effects; nevertheless, in order to preserve the properties of vaccinia unimpaired, it is prudent to renew it as frequently as possible from the cow. 6. Re-vaccination is the only means we possess of distinguishing the complete success of vaccinia from the less complete grades of protection. 7. Re-vaccination, however, is not certain evidence that the vaccinated in whom it had succeeded would have been destined to contract small-pox, but merely that it was probably among those that this latter malady was most likely to appear, if they became exposed to its infection. In ordinary times re-vaccination may be practised after fourteen years; during epidemic small-pox it may be practised after a shorter period.

